# Evaluating the use of posterior oropharyngeal saliva in a point-of-care assay for the detection of SARS-CoV-2

**DOI:** 10.1080/22221751.2020.1775133

**Published:** 2020-06-14

**Authors:** Jonathan Hon-Kwan Chen, Cyril Chik-Yan Yip, Rosana Wing-Shan Poon, Kwok-Hung Chan, Vincent Chi-Chung Cheng, Ivan Fan-Ngai Hung, Jasper Fuk-Woo Chan, Kwok-Yung Yuen, Kelvin Kai-Wang To

**Affiliations:** a Department of Microbiology, Queen Mary Hospital, Pokfulam, Hong Kong Special Administrative Region, People’s Republic of China; b State Key Laboratory for Emerging Infectious Diseases, Department of Microbiology, Carol Yu Centre for Infection, Li Ka Shing Faculty of Medicine, The University of Hong Kong, Pokfulam, Hong Kong Special Administrative Region, People’s Republic of China; c Department of Clinical Microbiology and Infection Control, The University of Hong Kong-Shenzhen Hospital, Shenzhen, People’s Republic of China; d Infection Control Team, Queen Mary Hospital, Hong Kong West Cluster, Pokfulam, Hong Kong Special Administrative Region, People’s Republic of China; e Department of Medicine, Li Ka Shing Faculty of Medicine, The University of Hong Kong, Pokfulam, Hong Kong Special Administrative Region, People’s Republic of China

**Keywords:** COVID-19, SARS-CoV-2, saliva, nasopharyngeal swab, point-of-care testing

## Abstract

During the Coronavirus disease 2019 (COVID-19) pandemic, logistic problems associated with specimen collection limited the SARS-CoV-2 testing, especially in the community. In this study, we assessed the use of posterior oropharyngeal saliva as specimens for the detection of SARS-CoV-2 in an automated point-of-care molecular assay. Archived nasopharyngeal swab (NPS) and posterior oropharyngeal saliva specimens of 58 COVID-19 patients were tested with the Xpert^®^ Xpress SARS-CoV-2 assay. SARS-CoV-2 was detected in either NPS or saliva specimens of all patients. Among them, 84.5% (49/58) tested positive in both NPS and saliva, 10.3% (6/58) tested positive in NPS only, and 5.2% (3/58) tested positive in saliva only. No significant difference in the detection rate was observed between NPS and saliva (McNemar’s test *p* = 0.5078). The detection rate was slightly higher for N2 (NPS 94.8% and Saliva 93.1%) than that of the E gene target (Saliva: 89.7% vs 82.8%) on both specimen types. Significantly earlier median Ct value was observed for NPS comparing to that of saliva on both E (26.8 vs 29.7, *p* = 0.0002) and N2 gene target (29.3 vs 32.3, *p* = 0.0002). The median Ct value of E gene target was significantly earlier than that of the N2 gene target for both NPS (26.8 vs 29.3, *p* < 0.0001) and saliva (29.7 vs 32.3, *p* < 0.0001). In conclusion, posterior oropharyngeal saliva and NPS were found to have similar detection rates in the point-of-care test for SARS-CoV-2 detection. Since posterior oropharyngeal saliva can be collected easily, the use of saliva as an alternative specimen type for SARS-CoV-2 detection is recommended.

## Introduction

Coronavirus disease 2019 (COVID-19) pandemic has caused significant impact on the healthcare system and socioeconomic activity. Early diagnosis is critical for prompt actions on patient management, infection control, and public health control measures [1]. Mass testing, together with rigorous contact tracing and quarantine and isolation, has been recommended to stop the pandemic [2].

An important logistic challenge to provide massive testing is specimen collection. Nasopharyngeal and oropharyngeal specimens are usually considered to have the highest diagnostic sensitivity and have been recommended by the World Health Organization. However, collecting nasopharyngeal and oropharyngeal specimens require significant manpower. Furthermore, there is currently a shortage of swabs in many places.

To solve the logistic problems associated with specimen collection, we need an easily collected specimen type. In a series of studies, we have demonstrated that posterior oropharyngeal saliva, collected by asking the patient to spit into a sterile bottle, has high sensitivities in the detection of respiratory viruses [3,4]. Posterior oropharyngeal saliva has been demonstrated to be useful in monitoring the viral load of patients with severe acute respiratory syndrome coronavirus 2 (SARS-CoV-2) infection [5,6]. Similar findings have also been demonstrated by other groups [7]. Because of the ease of sample collection, self-collected posterior oropharyngeal saliva has been used in Hong Kong for diagnostic testing at the out-patient clinic and at the accident and emergency department, screening of travelers at the airport, and contact tracing for COVID-19 cases [8].

Recently, automated systems for the rapid detection of SARS-CoV-2 are available. Three devices including Accula SARS-CoV-2 Test (Mesa Biotech, San Diego, CA), ID NOW COVID-19 (Abbott Diagnostics, Scarborough) and Xpert^®^ Xpress SARS-CoV-2 test (Cepheid, Sunnyvale, CA), have received US Food and Drug Administration (FDA) Emergency Use Authorization (EUA) as Clinical Laboratory Improvement Amendments (CLIA)-waived tests as of 10 May 10 2020 [9]. These systems not only shorten the turn-around-time for diagnostic tests, but also allow point-of-care testing (POCT). Currently, saliva specimens are not validated by the manufacturers for use in these automated systems. However, our previous study showed that saliva specimens can also be used as an automated system with a high sensitivity of detection of influenza viruses and respiratory syncytial virus [3]. In this study, we compared the use of NPS and posterior oropharyngeal saliva in a point-of-care assay for the detection of SARS-CoV-2.

## Materials and methods

### Sample collection

Fifty-eight pairs of archived nasopharyngeal swab (NPS) and posterior oropharyngeal saliva specimens collected from 58 COVID-19 positive inpatients in Queen Mary Hospital were included in this study. For each patient, NPS and saliva specimens were collected on the same day. NPS was collected using flocked swab and was immersed in 2 mL of viral transport medium as described previously [10]. Posterior oropharyngeal saliva was collected as described previously [3–6]. Briefly, patients were asked to cough up saliva by clearing the throat and spit about 1 mL of posterior oropharyngeal saliva directly into a sterile bottle in the early morning before mouth rinsing and breakfast, and 2 mL of viral transport medium was added to the saliva immediately. The samples were first tested with an in-house SARS-CoV-2 RNA dependent RNA polymerase/Helicase (RdRp/Hel) real-time RT–PCR assay [11]. This study has been approved by the Institutional Review Board of The University of Hong Kong/Hospital Authority Hong Kong West Cluster (UW 20-286).

### Point-of-care test

NPS and saliva specimens were tested by the Xpert Xpress SARS-CoV-2 assay (Cepheid, Sunnyvale, CA) according to manufacturer’s instruction. Briefly, 300 μL of each specimen in viral transport medium was directly loaded into the Xpert cartridge. The cartridge was loaded into the GeneXpert XVI system (Cepheid, Sunnyvale, CA). The assay targeted the E and N2 gene of SARS-CoV-2. Results were interpreted after 50 min run.

### Statistical analysis

Statistical analysis was performed using Medcalc 14.12.0 (MedCalc Software, Ostend, Belgium) and PRISM 6.0 (GraphPad Software, San Diego, CA). The diagnostic performance of using NPS and saliva was compared using McNemar’s test. The Ct values of NPS and saliva in point-of-care test were compared using Wilcoxon signed-rank test. For statistical analysis, specimens tested negative were assigned a Ct value of 46. A *p*-value of <0.05 was considered statistically significant.

## Results

This study included NPS and posterior oropharyngeal saliva from 58 patients. The median age was 38 years (interquartile range [IQR] 31–52 years), and 51.7% of them (30/58) were female. All 58 patients had either NPS or saliva tested positive by Xpert Xpress SARS-CoV-2 assay. Of these, 84.5% (49/58) tested positive in both NPS and saliva, 10.3% (6/58) tested positive in NPS only, and 5.2% (3/58) tested positive in saliva only. No significant difference in the detection rate was observed between NPS and saliva for the Xpert assay (McNemar’s test *p* = 0.5078) (Table 1). The results from Xpert assay had 100% concordance with our in-house RdRp-Hel RT–PCR.

**Table 1.** Comparison between the detection rate in NPS and saliva.

	NPS		
Saliva	Positive	Negative	Total
Positive	49 (84.5%)	3 (5.2%)	52 (89.7%)
Negative	6 (10.3%)	0	6 (10.3%)
Total	55 (94.8%)	3 (5.2%)	58 (100%)

When we compared the detection rate of the two gene targets, we found that the N2 gene target demonstrated slightly higher detection rate (NPS 94.8%; saliva 93.1%) than that of the E gene target (NPS: 89.7%; saliva: 82.8%). However, no significant difference was observed (McNemar’s test: NPS, *p* = 1.0; Saliva, *p* = 0.125).

**Figure 1.**
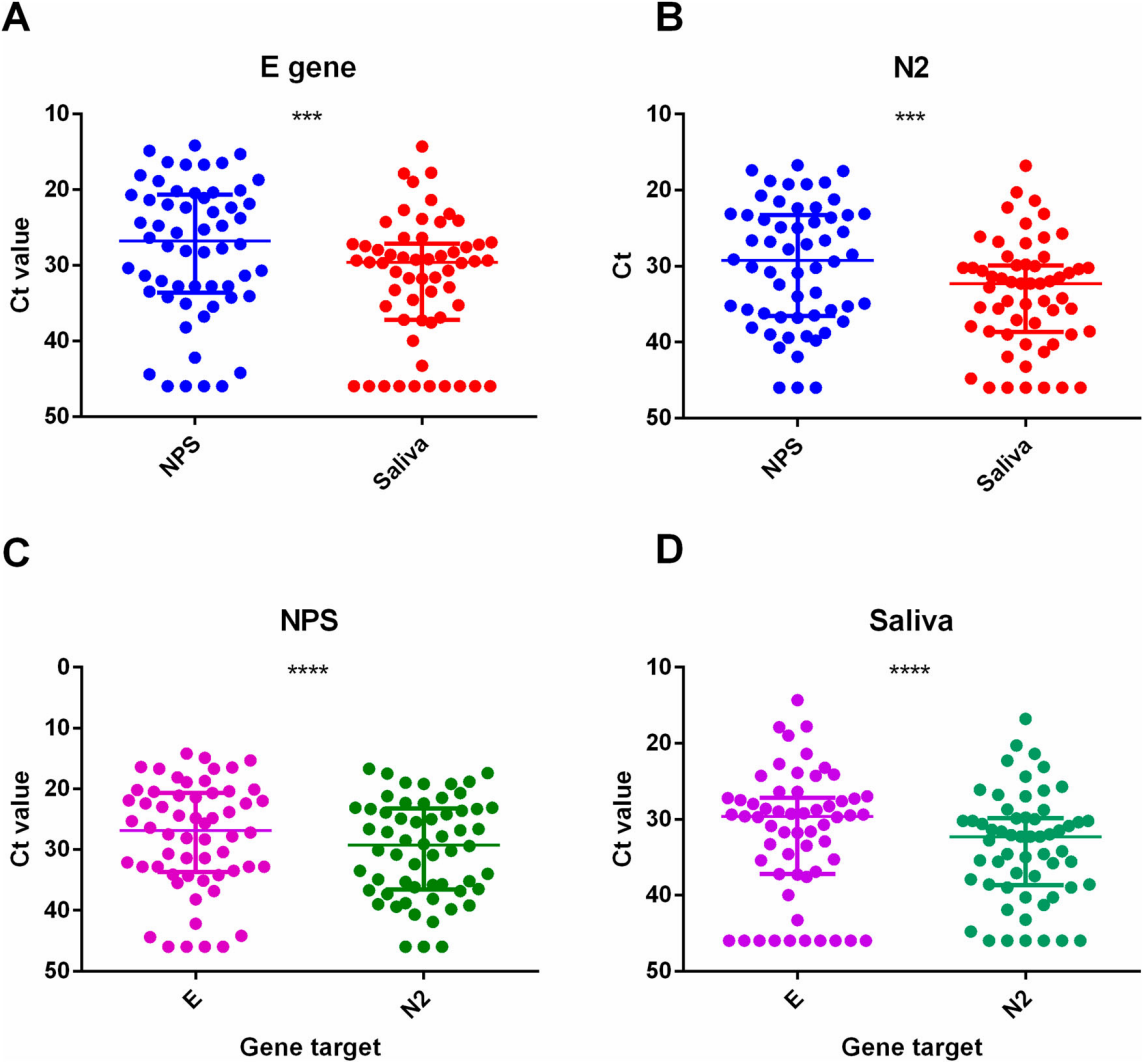
Comparison of Ct values. (A) and (B) Comparison of Ct values between NPS and saliva specimens for (A) E and (B) N2. (C) and (D) Comparison of Ct values between E and N2 for (C) NPS and (D) saliva.

For the Ct value difference between NPS and saliva, significant earlier median Ct value was observed for NPS comparing to that of saliva on both E (NPS: 26.8, IQR 20.7–33.5; saliva: 29.7, IQR 27.2–37.2; *p* = 0.0002) and N2 gene target (NPS: 29.3, IQR 23.3–36.5; saliva: 32.3, IQR: 29.9–38.6; *p* = 0.0002) (Figure 1A and B).

Next, we compared the Ct values of NPS between saliva-negative and saliva-positive patients. The median NPS Ct value of E gene target was significantly later for saliva-negative patients (38.2, IQR 31.9–44.3) than that of saliva-positive patients (25.1, IQR 20.4–32.8) (*p* = 0.0054). The median NPS Ct value for N2 gene target was also significantly later for saliva-negative patients (38.2, IQR 35.1–40.0) than that of saliva-positive patients (27.5, IQR 23.1–35.8) (*p* = 0.0057).

We also observed that the median Ct value of the E gene target was significantly earlier than that of the N2 gene target for both NPS (E: 26.8, IQR 20.7–33.5; N2: 29.3, IQR 23.3–36.5; *p* < 0.0001) and saliva (E: 29.7, IQR 27.2–37.2; N2: 32.3, IQR 29.9–38.6; *p* < 0.0001) (Figure 1(C,D)).

## Discussion

The detection results of the POCT on both NPS and saliva had 100% concordance to the results of our in-house real-time RT–PCR. We did not encounter any run failure during the nucleic acid extraction and point-of care test running process. Our study also demonstrated no significant difference in detection rates between NPS and saliva samples. Although discordant results were observed between NPS and saliva samples of 6 patients with relatively low viral load, both NPS and saliva tested positive for most of the patients. However, the median Ct values of both gene targets in NPS were significantly earlier than those in saliva. This is compatible with our previous results from other respiratory viruses, in which nasopharyngeal specimens had higher viral load than that in saliva specimens [3,4].

In comparing the performance between the gene targets of the Xpert assay, both the NPS and saliva specimens were found to have higher positive rate in N2 gene than in E gene. The N2 gene could detect >93% of samples in both NPS and saliva specimens, while the E gene could only detect <90% of NPS and saliva. This observation matched with the literature showing that the coronavirus N protein is abundantly produced within the infected cells [12–14].

If both NPS and saliva can provide similar detection rate in POCT, then the simplicity of sample collection will be critical. Collection of saliva is much simpler and easier than the collection of NPS. During the COVID-19 pandemic, the shortage of personal protective equipment and swab consumables supply affect the COVID-19 screening testing scale. Thus, the simple collection process of posterior oropharyngeal saliva should be an advantage. In addition, the simple procedures of the FDA EUA CLIA-waived assays such as the Xpert Xpress SARS-CoV-2 assay used in this study could further minimize the risk of processing error and shorten the total sample-to-reporting time.

To avoid bias in the timing of specimen collection, we assessed the viral load of saliva and NPS from the same patient on the same day to avoid bias due to different days of specimen collection. This is especially important because viral load on different days can vary. In our previous studies, we have demonstrated that SARS-CoV-2 viral load would gradually decline one week after symptom onset [5,6].

In conclusion, our study showed that posterior oropharyngeal saliva can be an alternative specimen type for SARS-CoV-2 detection in automated point-of-care system. The simple and easy sample collection procedure can even make it a better choice of specimen type during the COVID-19 pandemic.
